# AGING TREATMENT BY COUNTERACTING INTRINSIC DNA DAMAGE AND IMMUNOSENESCENCE

**DOI:** 10.1093/geroni/igz038.2990

**Published:** 2019-11-08

**Authors:** Andrei Gudkov

## Abstract

Progressive systemic poisoning by gradually accumulated damaged cells has been proposed as a major contributor to mammalian aging. Our preclinical studies support the hypothesis that this process results from a failure of innate immunity-mediated eradication of cells with DNA damaged by intrinsic mechanisms caused by the epigenetic desilencing of endogenous retroelements. This model suggests two translational approaches to improve the counteract accumulation of damaged cells: (i) by pharmacological suppression of LINE1 reverse transcriptase activities – the main driver of expansion of “retrobiome” and the trigger of damaged cell-associated inflammation and (ii) by counteracting immunosenescence by innate immunity stimulation. Preclinical proofs of concept have been obtained for both using nucleoside reverse transcriptase inhibitors and immunostimulators acting via TLR5 activation. Preparations for clinical testing of these agents in the context of age-related pathologies is in underway.

